# Protein identification by nanopore peptide profiling

**DOI:** 10.1038/s41467-021-26046-9

**Published:** 2021-10-04

**Authors:** Florian Leonardus Rudolfus Lucas, Roderick Corstiaan Abraham Versloot, Liubov Yakovlieva, Marthe T. C. Walvoort, Giovanni Maglia

**Affiliations:** 1 Groningen Biomolecular Sciences and Biotechnology Institute, University of Groningen, Groningen, Netherlands; 2 Stratingh Institute for Chemistry, University of Groningen, Groningen, Netherlands

**Keywords:** Peptides, Proteomic analysis, Single-molecule biophysics, Nanopores

## Abstract

Nanopores are single-molecule sensors used in nucleic acid analysis, whereas their applicability towards full protein identification has yet to be demonstrated. Here, we show that an engineered Fragaceatoxin C nanopore is capable of identifying individual proteins by measuring peptide spectra that are produced from hydrolyzed proteins. Using model proteins, we show that the spectra resulting from nanopore experiments and mass spectrometry share similar profiles, hence allowing protein fingerprinting. The intensity of individual peaks provides information on the concentration of individual peptides, indicating that this approach is quantitative. Our work shows the potential of a low-cost, portable nanopore-based analyzer for protein identification.

## Abstract (web summary)

Peptide mass fingerprinting is a traditional approach for protein identification by mass spectrometry. Here, the authors provide evidence that peptide mass fingerprinting is also feasible using FraC nanopores, demonstrating protein identification based on nanopore measurements of digested peptides.

## Introduction

The ever-increasing demand for high-throughput proteomic studies and personalized medicine requires the development of scalable and low-cost protein analysers^1–3^. Modern proteomics relies heavily on tandem mass spectrometry (MS) for its high precision and capability to identify and quantify proteins in complex mixtures^3–5^. However, most mass analyzers are large, have a high cost of investment, are expensive to maintain, and require specialized operators to function^2,3,6^.

In contrast to mass spectrometry devices, nanopore-based analyzers provide a low-cost and high-throughput platform with the adaptability towards native environments^7–10^. In nanopore analysis, analytes are measured as they disrupt an ionic current passing through individual nanopores under an applied potential. Importantly, the magnitude of the current blockade (I_B_) is mainly, altought not exclusively^11^, proportional to the volume the analyte excludes, allowing size discrimination of chemically similar (bio)polymers such as PEG chains, DNA, proteins, and peptides^12–24^. Furthermore, nanopores are capable of accurately detecting a variety of molecules, including proteins and DNA, with high precision at the single-molecule level^17,21,24–28^. Peptides are of special interest for protein characterization, as they allow identification analogous to bottom-up MS-based proteomics.

Three types of Fragaceatoxin C (FraC) nanopores FraC-T1, FraC-T2, and FraC-T3, most likely corresponding to octameric, heptameric and hexameric pores, respectively, can be used in peptide nanopore analysis^29^. FraC nanopores have been shown to differentiate peptides with a resolution of ~40 Da^29^, while also enabling the detection of small chemical modifications^30^. Recently we have shown that at acidic pH values (less than pH 4.5)^23^ peptides are captured by the nanopore despite their composition, and they are most efficiently analysed at the exact pH of 3.8^29^, the condition under which the nanopore has no significant electroosmotic flow^16^. Furthermore, we showed that the introduction of an aromatic residue in the sensing region of FraC (G13F-FraC containing a glycine to phenylalanine substitution at position 13) augmented the residence time of peptides inside the nanopore and their capture efficiency^16^.

**Fig. 1:**
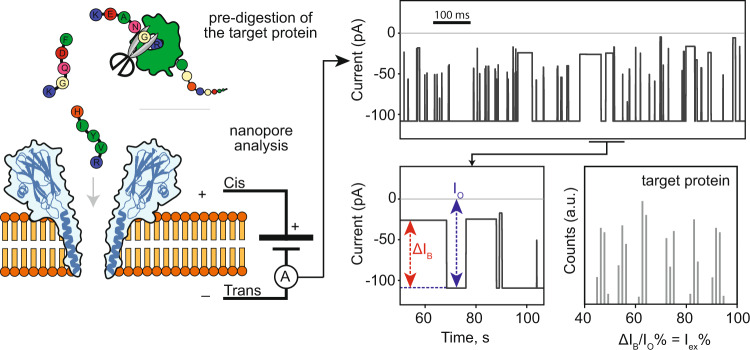
Graphical overview of the nanopore protein fingerprinting approach. Peptides are pre-hydrolyzed by a specific protease (e.g. trypsin) and the resulting peptides are measured as they translocate the nanopore. Each peptide entering the nanopore reduces the open pore current (I_o_) to the blocked pore current (I_B_). The resulting excluded current (ΔI_B_ = I_o_ − I_B_) relates to the volume of the peptide. The subsequent histogram of the percent of excluded currents (I_ex_ %= ΔI_B_/ I_O_ %) is used to identify the protein.

In this contribution, we show that G13F-FraC-T1 can be used to directly sample proteins that are digested by a protease. The resulting collected peptides describe a spectrum that can then be used to identify proteins (Fig. 1). A similar strategy relying heavily on the fingerprinting of (tryptic) peptides^5,22^ has been used in the early days of mass spectrometry for the detection and identification of proteins^31–33^. The nanopore approach might then provide a low-cost and high-throughput approach to protein identification. Furthermore, if the protease is implemented directly above the nanopore^34^, this approach is amenable for single-molecule identification.

## Results

### Calibration of the G13F-FraC-T1 nanopore

**Fig. 2:**
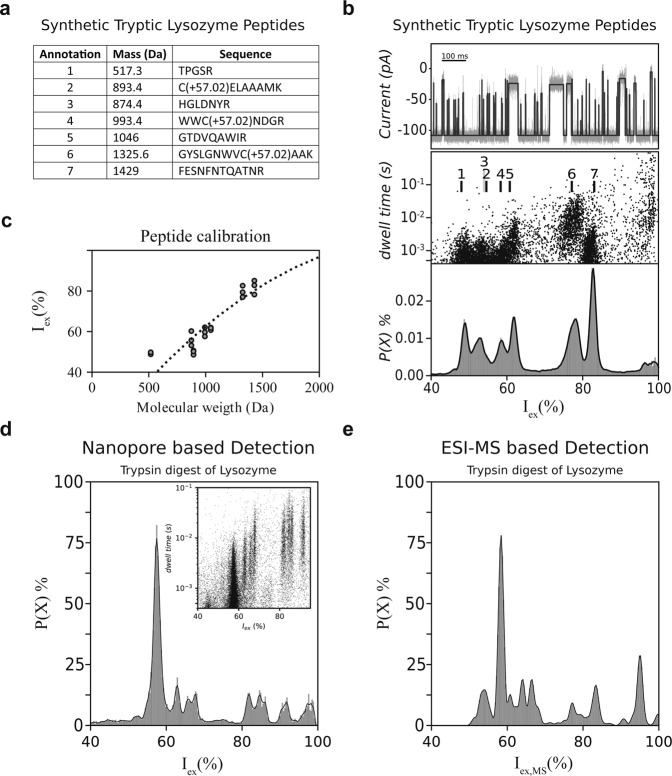
Lysozyme fingerprinting using FraC-G13F-T1 nanopores. **a** Peptides expected from lysozyme’s tryptic digest including their mass. The additional mass of 57.02 Da is expected for alkylated peptides at a cysteine position. **b** Ionic current versus time (top), I_ex_(%) versus dwell time (middle) and probability density (bottom) versus I_ex_(%) as obtained from the measurement of an equimolar mixture of the peptides expected from *Gallus-gallus* lysozyme. The black lines in the top panel indicate the fitted event. **c** Mass of model peptides from tryptic *Gallus-gallus* lysozyme (gray spheres) set against the measured excluded current (%), based on 3 individual measurements. The black dotted line represents a polynomial fit through the data. **d** Excluded current spectrum density from the tryptic digest of *Gallus-gallus* lysozyme. The inset shows the dwell time versus I_ex_(%) spectrum. **e** Constructed I_ex_% spectrum density from the same tryptic digest of *Gallus-gallus* lysozyme analysed by ESI-MS. Each peak indicates a peptide identified by ESI-MS, with I_ex_% calculated using the calibrated exclusion current % in **b**. The height of the peak reflects the relative abundance of peptides measured by ESI-MS. The spread of the peak indicates an arbitrary standard deviation of 0.5 I_ex_%. All nanopore measurements were performed in 1 M KCl buffered to pH 3.8 using 50 mM citric acid titrated with bis-tris-propane under an applied potential of −70 mV. Recording was performed at 50 kHz using an analog Bessel-filter at 10 kHz and a digital Gaussian filter of 5 kHz. Source data are provided as a Source Data file.

In order to characterize G13F-FraC-T1 for protein analysis, we first measured seven (synthetic) peptides (Fig. 2a) with a mass between 500 and 1700 Da that would have resulted from the complete, *in-silico*, hydrolysis of *Gallus-gallus* lysozyme. We included a reduction/alkylation procedure using dithiothreitol (DTT) and iodoacetamide (IAA) prior to nanopore analysis, to prevent disulfide bond formation interfering with the protein hydrolysis. The peptide signals were measured in 1 M KCl at pH 3.8 under an applied potential of −70 mV and recorded at 50 kHz filtered to 10 kHz using an analog Bessel-filter, and further processed using a digital Gaussian filter at 5 kHz (Fig. 2b). Under these conditions the peptides are expected to have one positive charge as the acidic residues should be mainly protonated at low pH. Notably, this is a recurring feature for trypsinated substrates, because trypsin cleaves preferentially after a lysine or arginine residue, thus most peptides will have a positive charge next to the C-terminus of the peptide, yielding an overall net charge of +1. Under these conditions, numerous peptide translocation events were observed, each with a specific current blockade (I_B_) (Fig. 2b). For each blockade, the percentage excluded current (I_ex_%) was calculated from the decrease in current observed during a blockade (ΔI_B_) relative to the observed current of the open pore (I_O_, Fig. 1). We show the dwell time and excluded current as well as a histogram of the excluded current (excluded current spectrum, or I_ex_% spectrum) of all seven synthetic peptides added cumulatively in equimolar concentrations (Fig. 2b). The signals corresponding to the individual peptides were further confirmed by assessing the peptides individually. Work with alpha-hemolysin, aerolysin, and FraC nanopores revealed that the relationship between the I_ex_% and mass of the analyte might be complex. Although the electrical signal relates primarily on the volume excluded by the analytes^29,35^, other factors such as the structure of the peptide, or the interactions of the peptides with the pore surface, or with the electrolyte and other buffer elements might play a role^17,20,29,30,36–42^. In a prior contribution, utilizing a wild type FraC nanopore, we described the relationship between the blockade and the mass of the peptides as a second order polynomial^29^. Here we also found that a second order polynomial allowed a reasonable fit (Fig. 2c), although an alternative fitting is possible (Supplementary Fig. 1)^38^. A better relationship will probably be obtained once the volume rather than the mass of the peptide will be accounted for.

### Detection of tryptic digested *Gallus-gallus* lysozyme

Next, we performed the experimental tryptic digest of *Gallus-gallus* lysozyme, and constructed an I_ex_% spectrum. The sample was alkylated and reduced using dithiothreitol/iodoacetamide, and digested using mass spectrometry-grade trypsin (Fig. 2d). We noticed that the smaller peptides, TPGSR (1), C(+57.02)ELAAMK (2), and HGLDNYR (3) were not clearly observed in the tryptic digest when measured using a nanopore. Electrospray ionization mass spectrometry (ESI-MS) confirmed that (1) and (2) were not present in the sample and we could therefore assume that this is a result of incomplete cleavage. Peptide (3) is detected less efficiently by the nanopore system compared to other peptides. Possibly, this is caused by the increased charge density caused by the presence of a histidine residue in the peptide sequence, which brings an additional positive charge (at pH 3.8) compared to other trypsinated peptides. In turn, for small peptides this might reduce the retention inside the nanopore by a stronger electrophoretic force.

In order to compare mass spectrometry and nanopore analysis, we converted the ESI-MS measurement into an expected I_ex,MS_% spectrum using an ad-hoc algorithm (Fig. 2e). Each peptide detected by ESI-MS was converted into a peak in the I_ex,MS_% spectrum, of which the specific position on the x-axis was extrapolated from the I_ex_% calibration curve from the individual lysozyme peptides (Fig. 2c). The spread (width) of the peak was introduced using an arbitrary standard deviation (*σ*) of 0.5 I_ex_%, while the height of the peak was derived from the relative peptide abundance from the ESI-MS measurement. Notably, we observed a good correlation between the nanopore measurements and ESI-MS-based mapping (Fig. 2e). As in the case of ESI-MS, it is expected that both positively charged and hydrophobic peptides are better captured by the nanopore than peptides lacking chemical functionalities. This is because the electrophoretic migration is augmented in positively charged peptides, while the retention of hydrophobic peptides is increased by the interaction with the hydrophobic inner surface of the G13F-FraC-T1 nanopore.

### Protein profiling using G13F-FraC-T1

**Fig. 3:**
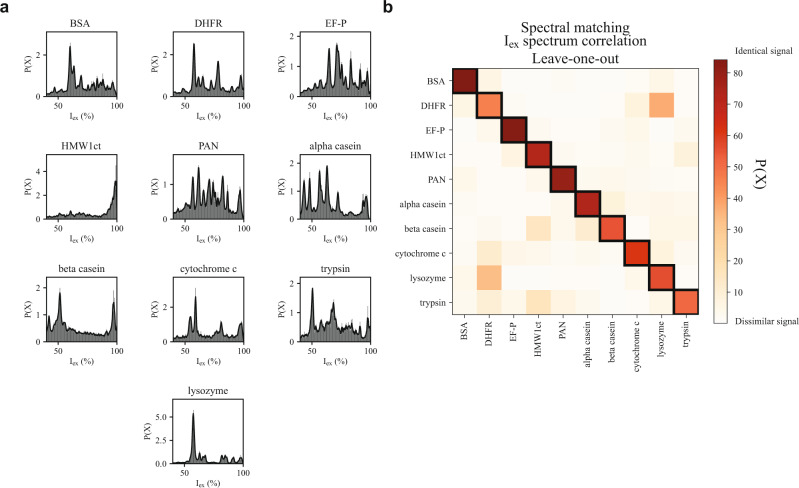
Protein identification using nanopore spectrometry. **a** Baseline-corrected excluded current spectra of 9 tryptic digested proteins. Based on 3 individual measurements. **b** Leave-one-out spectral matching of the baseline corrected excluded current spectra using Euclidean cosine cross-correlation. With on the Y-axis the protein that is being matched using leave-one-out matching with the proteins on the X-axis. The black boxes represent the matches with the highest score. All nanopore measurements were performed in 1 M KCl buffered to pH 3.8 using 50 mM citric acid titrated with bis-tris-propane under an applied potential of −70 mV. Recording was performed at 50 kHz using an analog Bessel-filter at 10 kHz and a digital Gaussian filter of 5 kHz.

To further test the approach of protein fingerprinting using nanopores, we selected nine additional proteins with a molecular weight between 12.4 and 66.5 kDa: cytochrome C (12.4 kDa), elongation factor P (EF-P, 21.0 kDa)^43^, dihydrofolate reductase (DHFR, 19.1 kDa)^44^, alpha casein (24.5 kDa), beta casein (25.1 kDa), bovine trypsin (trypsin, 23.3 kDa), C-terminal part of the high molecular weight adhesin 1 (HMW1ct, 34.6 kDa)^45^, proteasome-activating nucleotidase (PAN, 49.6 kDa), and bovine serum albumin (BSA, 66.5 kDa). For each protein, we prepared a tryptic digest and subjected the resulting peptide mixture to measurement with the FraC nanopore. From these experiments, we constructed an I_ex_% spectrum (Fig. 3a, Supplementary Figs. 2, 3, and 4) and related to the expected I_ex,MS_% built from ESI-MS (Supplementary Fig. 5) as well as the in-silico predicted peptide masses (Supplementary Fig. 6). Rewardingly, we find that the reproducibility of the spectra is very high (Supplementary Fig. 7). Comparison between the I_ex_% spectrum and the I_ex,MS_% revealed that the predicted I_ex,MS_% of BSA, DHFR, EF-P, PAN, trypsin, and lysozyme correlated well with the experimental nanopore data, while other proteins such as cytochrome C and alpha casein showed a less accurate prediction displaying more peaks than observed in the nanopore. Finally, beta-casein and HMW1ct, were poorly resolved in the nanopore. We noticed that not all the peaks overlapped between the I_ex_% and I_ex,MS_%, most likely because the I_ex,MS_% spectra are produced considering the mass of the peptides, while current blockades relate to the volume of the analyte. Hence, nanopore analysis might require further improvements, for example by using nanopores with higher resolution or by reducing the sample complexity using chromatography devices upstream of the nanopore measurements, as is already the case in LC-MS analysis. In order to test whether nanopores are capable of distinguishing each protein, we analysed the I_ex_% *spectra* using a spectral matching algorithm (see methods). Interestingly, we observed that all nine proteins and lysozyme are correctly assigned under these conditions (Fig. 3b), revealing that the nanopore approach can be used to identify proteins.

## Discussion

Proteins are commonly identified using bottom-up mass spectrometry (MS), where proteins are enzymatically digested at specific sites and the resulting peptides are identified^46^. MS analysis has revolutionized our understanding of proteins and almost single handedly initiated the emerging field of proteomics^3–5^. Although MS can now identify thousands of proteins in mixtures, some peptides escape MS detection and a relatively large amount of material is required. Therefore, addressing post translational modifications where only a few proteins are modified, heterogeneous proteins or low abundance proteins is challenging. Furthermore, mass spectrometers, are expensive and complicated devices that have high operational costs, which is why in protein analysis they are most often operated in centralized facilities^2,3,6^. In this work we introduce a method for protein analysis based on nanopores. Unlike MS, the electrical output signal arising from a nanopore can be easily interfaced with low-cost and portable devices. Nanopores have additional advantages as they detect single-molecules and they are amenable to incorporation into arrays of thousands of nanopores for high throughput analysis. However, despite nanopores having been applied successfully to sequence DNA, their use in protein identification has yet to be proven.

In this contribution, we describe a nanopore approach for the identification of proteins. Using FraC nanopores, we show that the tryptic digest of proteins generates clustered events from individual trypsinated peptides. Calibration using synthetic peptides corresponding to the expected complete hydrolysis of *Gallus-gallus* lysozyme (mass between 500 and 1700 Da) revealed that the nanopore captured all peptides and that an approximate relationship between the signal and the size of the peptide can be established (Fig. 2). Comparison between ten protein substrates showed a reasonably good correlation with MS analysis (Fig. 2 and Supplementary Fig. 5), and revealed that proteins can be recognized by spectral matching, which is similar to peptide mass fingerprinting (PMF), a technique used in early protein analysis. Although the proteins tested here are very different from each other, previous work showed that nanopore currents can detect differences in one single amino acid^20,38,39,42^ or single post-translational modifications^30,47^. At present, we observe misassignments between peptide masses, which complicates the identification of unknown peptides and makes the recognition of unknown proteins challenging. However, nanopore currents report the volume of a peptide rather than its mass. Hence, we expect large improvements once calibration curves that take into consideration the exact volume of the peptide in solution will be used. We also found that peptide abundance measured by the nanopore matched relatively well that predicted by MS. Further research on the relationship between the capture frequency and the physico-chemical properties of the peptides will reveal the strength and limitation of nanopore analysis for quantification of peptides.

At the moment, the resolution of nanopore analysis does not yet match that of mass spectrometry. However, improvements can be envisaged. For example, upstream separation devices could be used, the interaction between the nanopore and the analytes can be improved, or nanopores with different sizes, shapes and physical properties can be used. Nonetheless, nanopores have distinguishable advantages: they can be integrated into inexpensive and portable devices^1,3^, they work in solution and they can be directly interfaced with other analytical devices such as liquid chromatography or spectroscopy devices. Further, nanopores measure a fundamentally different property of peptides compared to MS (volume rather than mass/charge). Hence, nanopores might be used alongside MS for the identification and/or quantification of analytes (*e.g*. isobaric peptides) that are not easily studied by MS. Furthermore, arrays of thousands of nanopores are currently under development in commercial devices, suggesting that nanopore arrays have the potential to allow high-throughput protein analysis. Finally, nanopores are single-molecule sensors. Therefore, if a protease will be coupled to a nanopore directly^34^, this approach might be used for fingerprinting-based identification of single molecules. The latter might find application in the characterization of low abundance proteins or the heterogeneity of protein samples.

## Methods

### Chemicals

Potassium chloride, sodium chloride, urea, imidazole, *N,N*-Dimethyldodecylamine *N*-oxide (LDAO), n-Dodecyl β-D-maltoside (DDM), and lysozyme were received from Carl-Roth. 2-Amino-2-(hydroxymethyl)−1,3-propanediol (Tris) was obtained from Roche; citric acid, bovine serum albumin (BSA), and n-hexadecane were purchased from Acros. Pentane, magnesium chloride, dithiothreitol, iodoacetamide, trypsin, beta-casein, cytochrome C, alpha-casein, sheep blood and peptides were purchased from Sigma-Aldrich (Merck), ethanol from (Boomlab). 1,2-diphytanoyl-*sn*-glycero-3-phosphocholine (DPhPC), and sphingomyelin were received from Avanti Polar Lipids. Ni-NTA beads were obtained from Qiagen.

### Protein digestion

In total 100 µg of protein stock was taken and the volume was adjusted to 50 µl using 20 mM Tris.HCl buffer (pH 7.5). A final concentration of 20 mM dithiothreitol (DTT) was added to reduce any disulfide bonds. The sample was incubated at 37 °C for 15 min followed by a denaturing step at 95 °C for 15 min. Afterwards, a 20 mM iodoacetamide (IAA) was added and the sample was left to incubate for 15 min at room temperature in the dark in order to alkylate the reduced cysteine residues. Finally, the total volume was adjusted to 100 µl using 100 mM Tris Buffer (pH 8.5).

Tryptic digestion was performed using a kit purchased from Sigma-Aldrich, containing proteomics grade trypsin singles. A total of 50 µl of sample (containing 50 µg of protein) was added to 1 µg of mass-spec grade trypsin (1:50 enzyme:protein ratio) and the sample was subsequently incubated overnight at 37 °C. Since large (>>2000 Da) peptides could clog the nanopore, the protein solution was passed through a centrifugal filter with a molecular weight cut-off of 3000 Da (Amicon). Filtered samples were stored at −20 °C prior to use.

### Fragaceatoxin C monomer purification

Fragaceatoxin C nanopores were expressed and purified as described previously^26^. In brief, a pT7-SC1 plasmid containing the G13F-*FraC* gene (Supplementary Table 1), was electrochemically transformed into BL21(DE3) competent *Escherichia coli* cells and grown overnight at 37 °C on LB agar plates supplemented with 100 mg/L ampicillin and 1% glucose. On the next day, grown LB plates were solubilized into 200 mL 2xYT medium, supplemented with 100 mg/L ampicillin. Cultures were grown under constant shaking at 37 °C until an optical density (OD_600_) of 0.6 was reached. Afterwards, 0.5 mM isopropyl β-D-1-thiogalactopyranoside was added for induction and growth continued overnight at 21 °C. Bacterial cells were pelleted using centrifugation (6,000 *g*, 20 min, 4 °C) and stored for at least one hour at −80 °C. 100 mL (original culture) cell pellets were resuspended using 20 ml lysis buffer, consisting of 150 mM NaCl buffered at pH 7.5 using 15 mM Tris base and supplemented with 1 mM MgCl_2_, 2 M Urea, 0.2 mg/mL lysozyme, 0.2 units/mL DNase and 20 mM imidazole. Solubilised pellets were vigorously shaken for 1 hour at room temperature. The lysate was disrupted fully by sonification for 60 s (duty cycle 30%, output control 3) using a Branson Sonifier 450. The lysate was centrifuged at 6000 *g* for 20 min at 4 °C and the supernatant was transferred to a fresh 50 mL falcon tube. Subsequently, 200 µL pre-washed Ni-NTA beads (Qiagen, stored at 4 °C) was added and the falcon tube was incubated for 1 h at room temperature (21 °C) under constant rotation. The supernatant, incubated with beads, was added to a pre-washed Micro Bio-Spin column (Bio-Rad) and, afterwards, washed extensively using a buffer consisting of 150 mM NaCl buffered at pH 7.5 using 15 mM Tris base supplemented with 20 mM imidazole. The column was dried by centrifugation (500 *g*, 1 min) in order to remove residual wash buffer. Finally, monomeric protein was eluted (500 *g*, 2 min) after a 10 min incubation (room temperature, 21 °C) with 150 µL of 150 mM NaCl buffered at pH 7.5 using 15 mM Tris base and supplemented with 300 mM imidazole.

### Sphingomyelin-DPhPC liposomes preparation

An equal mixture of 25 mg 1,2-diphytanoyl-*sn*-glycero-3-phosphocholine (DPhPC) and 25 mg sphingomyelin (Brain, Porcine) was dissolved in 4 mL pentane containing 0.5 v/v% ethanol. A film was formed on the side of a round bottom flask by application of heat under constant rotation, evaporating all solvents. The resulting film was dissolved in 10 mL of 150 mM NaCl, buffered at pH 7.5 using 15 mM Tris base. The resulting liposome solution (5 mg/mL) was frozen (−20 °C) and thawed multiple times.

### Fragaceatoxin C oligomerisation

Freeze-thawed liposomes were added to purified monomers of fragaceatoxin C in a mass ratio of 10:1 (liposomes:protein). Liposomes were left to incubate for 30 min, at 37 °C, and later solubilised by the addition of 0.6 v/v% LDAO. Subsequently, the solution was diluted 20 times with 150 mM NaCl buffered at pH 7.5 using 15 mM Tris supplemented with 0.02% DDM. 200 µL washed regenerated Ni-NTA were added and incubated for 1 h at room temperature (21 °C) under constant rotation.

Incubated Ni-NTA beads were transferred onto a pre-washed Micro Bio-Spin column (Bio-Rad) and washed extensively using a buffer consisting of 150 mM NaCl buffered at pH 7.5 using 15 mM Tris base supplemented with 20 mM imidazole and 0.02 v/v% DDM. The Micro Bio-Spin column was dried by centrifugation (500 *g*, 1 min) in order to remove residual wash buffer. Protein was eluted (500 *g*, 2 min) after a 10 min incubation (room temperature, 21 °C) with 150 µL of 150 mM NaCl buffered at pH 7.5 using 15 mM Tris base supplemented with 1 M imidazole and 0.02 v/v% DDM. The oligomers were stored at −80 °C for long-term storage, thawed aliquots were kept at 4 °C and refreshed regularly.

### Plasmid preparation

DHFR: A pT7-SC1 plasmid^48^ containing the His_6_-tagged *DHFR* gene (Supplementary Table 1), available from a previous study^13^. PAN: A synthetic gene containing His_6_-tagged *PAN* (obtained from Integrated DNA Technologies, Supplementary Table 1) was ligated into a pT7-SC1 plasmid. HMW1ct: A pET45b plasmid harboring the *hmw1ct* gene (Supplementary Table 1) was constructed as described previously^49^, EF-P. A pBAD-His_6_-SUMO plasmid harboring the *efp* gene (from *Pseudomonas aeruginosa* PAO1, synthesized and cloned by GenScript, Supplementary Table 1).

### Expression of dihydrofolate reductase (DHFR) and proteasome-activating nucleotidase (PAN)

Plasmids containing the gene of interest were electrochemically transformed into BL21(DE3) competent *Escherichia coli* cells. The cells were grown overnight at 37 °C on LB agar plates supplemented with 100 mg/L ampicillin and 1% glucose. On the next day, grown LB plates were solubilized into 200 mL 2xYT medium, supplemented with 100 mg/L ampicillin. Cultures were grown under constant shaking at 37 °C until an optical density (OD_600_) of 0.6 was reached. Afterwards, 0.5 mM isopropyl β-D-1-thiogalactopyranoside was added for induction and growth was continued overnight at 21 °C. Cells were pelleted using centrifugation (6000 g, 20 min, 4 °C) and stored for at −80 °C.

### His_6_-tag protein purification of DHFR and PAN

Cell pellets from 100 mL culture were resuspended using 20 mL lysis buffer, consisting of Sdex (150 mM NaCl, 15 mM Tris at pH 7.5) supplemented with 1 mM MgCl_2,_ 0.2 mg/mL lysozyme, 0.2 units/mL DNase and 20 mM imidazole. Solubilised pellets were vigorously shaken for 40 min at room temperature. The lysate was disrupted fully by sonification using a Branson Sonifier 450. The lysate was centrifuged at 6000 g for 20 min at 4 °C and the supernatant was incubated with 200 µL pre-washed Ni-NTA beads (Qiagen) for 30 min at room temperature (21 °C) under constant rotation. The supernatant, incubated with beads, was added to a pre-washed Micro Bio-Spin column (Bio-Rad) and washed with Sdex supplemented with 20 mM imidazole. Finally, protein was eluted in steps of 150 µL with Sdex supplemented with 300 mM imidazole. The collected protein fractions were stored at −20 °C until analysed.

### Additional purification of PAN

Following His_6_-tag protein purification, in order to increase the purity, an additional purification step was included. Four fractions (600 µl) with the highest protein concentration were combined and purified using the anion exchange chromatography purification described in these methods. The presence of PAN was demonstrated by SDS-PAGE and the fractions with highest protein concentration were combined and concentrated using a 10 kDa MWCO spin filter (Amicon).

### Expression and purification of HMW1ct

A pET45b plasmid harboring the *hmw1ct* gene, constructed as described previously^49^, was used for expression of the HMW1ct protein (C-terminal fragment of *Haemophilus influenzae* high-molecular weight adhesin protein, residues 1205–1536). Cells were harvested by centrifugation (3220 g), resuspended in ice-cold lysis buffer (50 mM HEPES, 100 mM NaCl, 10% glycerol, pH 7.5), and lysed by sonication (Branson Sonifier 450: 30% duty cycle, 2.5 min) in the presence of the protease inhibitors cocktail (Roche). Cell debris was removed by centrifugation and supernatant was used for Ni-affinity chromatography purification. Briefly, 3–4 mL of Ni-NTA resins (Qiagen) were applied on the gravity column, washed with water, and equilibrated with the lysis buffer. Cell-free extract was then mixed with the resins for 1.5 h at 4 °C with gentle shaking. Afterward, cell-free extract was allowed to flow through and resin-bound proteins were washed twice with washing buffer (50 mM HEPES, 300 mM NaCl, 5% glycerol, 15 mM imidazole, pH 7.5) and then eluted in three steps with elution buffer (50 mM HEPES, 300 mM NaCl, 5% glycerol, 400 mM imidazole, pH 7.5). Fractions containing protein of interest were collected and dialyzed using SnakeSkin dialysis system (MWCO 10 kDa, Thermo Fischer Scientific) against storage buffer (50 mM HEPES, 100 mM NaCl, 10% glycerol, pH 7.5). After dialysis protein was aliquoted and stored at −80 °C until further use.

### Expression and purification of EF-P

A pBAD-His_6_-SUMO plasmid harboring the *efp* gene was used to transform chemically competent *E. coli* TOP10 cells (standard heat-shock protocol) and plated on LB-agar plates containing ampicillin. A single colony was selected from the plate and used to prepare glycerol stock. To express the protein on large scale, a preculture (10 mL) in LB (100 µg/mL ampicillin) was prepared from the glycerol stock and grown at 37 °C with shaking (200 rpm) for 16–18 h. The preculture was then used to inoculate 500 mL of Terrific Broth (TB) (100 µg/mL ampicillin) at 1:200 dilution ratio and incubated at 37 °C with shaking until OD_600_ reached values of 0.6–0.7. Protein expression was induced by addition of 0.05% l-Ara (w/v, final concentration) and further incubation for 4 h at 37 °C with shaking. Cells were harvested by centrifugation at 3220 g for 15 min (Sorvall centrifuge, F-12 6 × 500 LEX fixed angle rotor, Thermo Scientific). The supernatant was discarded, and the cell pellet was resuspended in ice-cold lysis buffer (20 mM Tris, 500 mM NaCl, pH 8) in the presence of the protease inhibitor cocktail (Roche, complete, EDTA-free). Cells were lysed by sonication (Branson Sonifier 450, output control 30%, 2 min) and subsequently spun down at 6311 g at 4 °C for 1 h. For His_6_-tag protein purification, the cell-free extract was incubated with Ni-NTA resin (Qiagen) for 1.5 hours at 4 °C with gentle shaking. The mixture was loaded on a gravity column and the lysate was allowed to flow through, followed by a washing step (twice) with washing buffer (20 mM Tris, 500 mM NaCl, 25 mM imidazole, pH 8). The protein of interest was eluted with elution buffer (20 mM Tris, 500 mM NaCl, 400 mM imidazole, pH 8) in three steps. Column fractions were analyzed by 12% SDS-PAGE analysis and the resulting gels were stained using Instant Blue protein stain. Fractions containing protein of interest were pooled and desalted using midi PD-10 desalting columns (GE Healthcare). The target protein was routinely obtained in yields of 20-40 mg per 1 L of culture. To cleave the His_6_-SUMO tag off, EF-P- His_6_-SUMO was incubated with SUMO-protease (1 mg SUMO-protease for 12.5 mg of EF-P- His_6_-SUMO) overnight at 4 °C. Next day, cleaved EF-P was purified with Ni-affinity chromatography. Briefly, reaction mixture was incubated with Ni-NTA resin for 1.5 h at 4 °C with gentle shaking. The resulting suspension was allowed to pass through the gravity column and the flow through was collected. Next, the resin was washed with washing buffer 1 (20 mM Tris, 500 mM NaCl, 15 mM imidazole, pH 8), and washing buffer 2 (20 mM Tris, 500 mM NaCl, 30 mM imidazole, pH 8). This was followed by elution with elution buffer (20 mM Tris, 500 mM NaCl, 400 mM imidazole, pH 8). Analysis of the purification fractions with SDS-PAGE indicated that most of EF-P protein was present in the flow-through fraction. This fraction was concentrated and stored at −80 °C until further use.

### Protein purification of bovine serum albumin (BSA)

A total of 10 mg lyophilised BSA (Arcos Organics) was dissolved in 1 mL of buffer A (50 mM Tris, pH 7.5) and purified using the anion exchange chromatography purification described in these methods. The presence of BSA was demonstrated by SDS-PAGE and the fractions with highest protein concentration were combined and concentrated using a 10 kDa MWCO spin filter (Amicon).

### Anion exchange chromatography purification

BSA and PAN were selected for subsequent purification using ÄKTA pure chromatography (GE Healthcare Life Sciences), equipped with an HiTrap Q HP anion exchange column (GE Healthcare Life Sciences). Samples were loaded onto the column using a flow of 1 mL/min buffer A (50 mM Tris, pH 7.5). Proteins were eluted using a flow of 1 mL/min with a 40% gradient of buffer B (1 M NaCl, 50 mM Tris, pH 7.5) in 40 min.

### Planar lipid bilayer electrophysiological recordings

A 25 µm thick Teflon membrane (Goodfellow Cambridge Ltd.) containing an aperture with a diameter of 100 µm was used to separate two compartments of a flow cell. 5 µL of a solution of 5% hexane in pentane (v/v) was applied near the aperture of the Teflon membrane. After an evaporation period of 1 min, 400 µL buffer, consisting of 1 M KCl buffered at pH 3.8 using 50 mM citric acid with bis-tris-propane, was added to both compartments of the chamber. Afterward, 20 µL of a 6.25 mg/mL solution of DPhPC in pentane was added on top of each compartment. The pentane was left to evaporate for ~2 min before mixing. A silver/silver chloride electrode was attached to each compartment. The Langmuir-Blodgett method, as described by Maglia et al.^23^, was used to create planar lipid bilayers. Peptides and protein digests were always added to the *cis* compartment of the chamber.

### ESI-MS experiments

For each trypsin-digested protein, ~10 µg is taken for mass-spectrometry analysis. The sample is analyzed with an LC system, EASY-nLC II (Thermo Scientific), connected to an LTQ Orbitrap XL (Thermo Scientific) using electron spray ionization (ESI). Peptides were separated in a reverse phase over an in-house packed C18 (ReproSil-Pur C18-AQ, 3 µm resin, Dr. Maisch) nano LC column (75 µm I.D., 15 cm, New Objective) under a gradient of solvent A: 2% acetonitrile, 0.1% Formic acid and solvent B: 0.1% formic acid in acetonitrile with 5% B to 28% B for 60 min, followed by 28 to 40% B for 10 min, 40 to 50% B for 2 min and 50% isocratic for 18 min at a flow of 200 nL/min and a column oven temperature of 60 °C. Prior MS analysis, the sample was cleaned using C18 tips (Pierce) to remove salts and other contaminants. From the mass-spectrometry measurements we obtain the peak area of the detected peptides, which we use to generate an I_ex_% spectrum.

### Data recording

High impedance ionic current recordings were obtained using an Axopatch 200B amplifier combined with a Digidata 1440a or Digidata 1550B A/D converter (Molecular Devices), similar to preceding work^23^. Data were recorded using Clampex 10 (Molecular Devices) at a sampling frequency of 50 kHz using an analog Bessel filter of 10 kHz, unless stated otherwise.

### In-silico digestion of lysozyme by trypsin

In-silico protein trypsination was performed from the single-letter code sequence by cleaving each arginine (R) and lysine (K) residues unless they were followed by proline (P) using (python) regular expression “.(?:(?<![KR](?!P)).)*“. Peptides with a mass lower than 500 Da and larger than 1700 Da were excluded, as they are not observed by the nanopore.

### Event and I_ex_% extraction

Peptide translocation events were extracted from the data using a threshold-search algorithm and characterized using a generalized flat-top normal distribution function (gNDF).

We use a threshold function to determine all events at 3σ from the baseline, and subsequently fit a generalized flat-top normal distribution. This distribution is a good descriptor for the events we observe, as it resembles a spike-like profile when *β* < 1, a Gaussian profile when *β* = 1, and a flat-top shape when *β* > 1. We filter events where *β* < 1, as these resemble a spike and the residual current (height) cannot be accurately estimated. Therefore, the events that we use to construct the residual current spectrum are all Gaussian or flat-top shaped, resulting in less variance between spectra.

This approach shares similarities with other methods to characterize events, such as MOSAIC^50,51^. In both cases, an idealized event are represented and a filter effect is applied. Advantageously, the gNDF describes both the filter effect and flat-top profile in a closed form. This allows the discrimination based on the event shape, however, it is slower for event recognition than using the algorithms implemented in MOSAIC.

1 $$f(x)=\Delta {I}_{B}\ast \exp \left(-{\left(\frac{{(x-\mu )}^{2}}{2{\sigma }^{2}}\right)}^{\beta }\right)+{I}_{O}\;{{{{{\rm{for}}}}}}\;\beta\, > \, 0$$

where *µ* is the events center in the time domain with variance *σ*^2^ and ΔI_B_ is the current difference (pA) between the baseline (I_O_) and the event maximum. The variable *β* describes the shape of the function.

We utilize the generalized flat-top normal distribution, which is also used for the estimation of the dwell time. We estimate the events based on the full-width at half maximum of this distribution. Therefore, the dwell time is not skewed in short events. We discard spike-like events with a *β* < 1, however, we would not be able to determine the dwell time of these events in either algorithm.

2 $$FWHM = 2\sigma\sqrt{2 \root \beta \of {{{{{{\mathrm{ln}}}}}}\,{2}}}$$

where *σ* equals the square root of the variance (*σ*^*2*^) and *β* describes the shape parameter.

For each event, the excluded current was calculated by dividing the median current difference between the blockade (I_B_) and the median open pore current (I_o_) by I_o_.

3 $${I}_{ex} \% =\frac{{I}_{B}-{I}_{o}}{{I}_{o}}\,\ast\, 100 \% =\frac{\Delta {I}_{B}}{{I}_{o}}\,\ast\, 100 \%$$

Where I_B_ is the blocked pore current, I_o_ represents the open pore current, and I_ex_% is the excluded current.

### I_ex_% spectrum construction and re-alignment

For each event, the I_ex_% was calculated and a histogram between 0 and 100 I_ex_% was constructed with a bin-width of 1 I_ex_%. The resulting histogram is called the excluded current spectrum, or abbreviated as the I_ex_% spectrum.

In order to correct for shifting in the residual current due to experimental fluctuations (*e.g*. slight differences in salt concentration, temperature, and instrument offset), we performed spectral re-alignment. We selected one spectrum (per protein) as a reference to which we re-align. Then, we subtracted bin-by-bin each additional spectrum to the reference spectrum and collected the residual sum of squares (as “error”). The x-axis of each bin of the new spectrum was moved in steps of 0.05 I_ex_% and the error recorded. After performing 100 step-wise additions (total +5 I_ex_%) and 100 step-wise subtractions (total −5 I_ex_%) a plot of ΔI_ex_% versus the error was obtained showing the spectral offset where the error is minimal (Supplementary Fig. 3).

### Data analysis

All the data were analysed using Python 3.7 and is contained within a Jupyter notebook as an ad-hoc script and is available from the corresponding authors upon reasonable request and at 10.5281/zenodo.5205565.

### Mapping mass to excluded current

When the average I_ex_% is plotted versus the peptide mass, we obtain a non-linear relationship, instead of a linear curve that would be expected if the excluded current would only depend on the mass of the peptide. It is important to notice that the observed current is reduced because of the steric exclusion of ions induced by the analyte. Steric exclusion correlates to the mass of a peptide due to the sum of partial volume^52^, and assumes no 3-dimensional structure, nor an interaction with the nanopore. We reasoned that the correlation between the molecular weight and I_ex_% of peptides can be represented as a second order polynomial (Eq. (4)^29^. We have shown this relationship in a previous contribution, and also include the origin in the fit. We used the poly1d function as implemented in the Numpy library to find the ideal fit around the data. We found the following parameters: b_2_ = −1.33 × 10^−5^, b_1_ = 7.23 × 10^−2^, and b_0_ = 3.28.

4 $${I}_{ex,MS}(m)={b}_{0}+{b}_{1}\,\ast\, m+{b}_{2}\,\ast\, {m}^{2}$$

where b_0_, b_1_, and b_2_ are the exponential terms. The input variable *m* represents the mass of the peptide.

### I_ex_*%* spectrum density from MS analysis

The mass of each peptide was converted to I_ex,MS_% using the fit of Fig. 2c. Each value was then used as the center (*µ*) a peak. The spread of each peak was made using an arbitrary standard deviation (*σ*) of 0.5 I_ex_%. The amplitude (*a*) of the peaks was matched to the area observed from ESI-MS measurements. Throughout this contribution, we utilized a ESI-MS containing an Orbitrap detector. The intensity of each ion is therefore measured as the amplitude of the free induction decay, which has a square relationship with the number of ions detected. Therefore, we had to take the square root of the intensity resulting in Eq. (5).

5 $$g(x)=\sqrt{\mathop{\sum }\limits_{i=0}^{n}{a}_{i}\ast \exp \left(-\frac{{(x-{\hat{I}}_{ex}({m}_{i}))}^{2}}{2{\sigma }^{2}}\right)}$$

where *a*_*i*_ is the area resulting from ESI-MS. *m*_*i*_ is the mass of the peptide and is mapped to the excluded current using Eq. (4). *σ* is the peak width.

### Spectral matching

We analyzed the I_ex_% *spectra* using a spectral matching algorithm incorporating the squared first derivate Euclidean cosine correlation (DEuc) (Eq. (6); which is advantageous, as it corrects for baseline sloping^53^. The DEuc is a direct result of the dot product equation and allows the estimation of the angle between two vectors, which can be used as a measure of similarity. We use the normalized counts of the excluded current spectra which are represented as vectors, *e.g*. $\mathop{{{{{{\boldsymbol{A}}}}}}}\limits^{\rightharpoonup }$ = [*a*_*1*_*, a*_*2*_*,…,a*_*n*_] and $\mathop{{{{{{\boldsymbol{B}}}}}}}\limits^{\rightharpoonup }$ = [*b*_*1*_*, b*_*2*_*,…,b*_*n*_]. We estimate the derivative of $\mathop{{{{{{\boldsymbol{A}}}}}}}\limits^{\rightharpoonup }$ and $\mathop{{{{{{\boldsymbol{B}}}}}}}\limits^{\rightharpoonup }$ by numerical differentiation, resulting in ${{{{{\boldsymbol{\Delta }}}}}}\mathop{{{{{{\boldsymbol{A}}}}}}}\limits^{\rightharpoonup }$ and ${{{{{\boldsymbol{\Delta }}}}}}\mathop{{{{{{\boldsymbol{B}}}}}}}\limits^{\rightharpoonup }$. Usage of the derivative is advantageous as this is less sensitive to background noise, which is usually stochastically distributed.

6 $$DEuc=\,\cos (\theta )=\frac{\Delta \mathop{A}\limits^{\rightharpoonup }\,\cdot \Delta \mathop{B}\limits^{\rightharpoonup }}{\Vert \Delta \mathop{A}\limits^{\rightharpoonup }\Vert \Vert \Delta \mathop{B}\limits^{\rightharpoonup }\Vert }=\frac{{({\sum }_{i=0}^{N}\Delta {a}_{i}\Delta {b}_{i})}^{2}}{{\sum }_{i=0}^{N}\Delta {a}_{i}^{2}\,\ast\, {\sum }_{i=0}^{N}\Delta {b}_{i}^{2}}$$

We chose to only consider the I_ex_% *spectrum* between 50 and 98 I_ex_%, as noise below the limit-of-detection and fully blocked events may have skewed the comparison. Subsequently, we performed a leave-one-out comparison using the DEuc as a score, normalised to 100% for visualisation. The leave-one-out comparison compares the I_ex_% spectrum of each measurement (sample), with the average I_ex_% spectrum of each protein (database). For each comparison, we constructed a database that contains all measurements except for the sample. The score represented in Fig. 3b of the main text are the average scores over all samples for each protein.

### Reporting summary

Further information on research design is available in the Nature Research Reporting Summary linked to this article.

## Supplementary information

Supplementary Information

Reporting Summary

## Data Availability

All data and corresponding analysis generated in this study have been deposited in the Zenodo database under 10.5281/zenodo.5205565. Source data underlying Fig. 2b, c and Supplementary Fig. 1 are provided as a Source Data file. Source data are provided with this paper.

## Source data

Source Data

## Code availability

All custom code and algorithms used in this study are available at 10.5281/zenodo.5205565.
