# Supplementary material for: Protein identification by nanopore peptide profiling
Source: Nat Commun. 2021 Oct 4;12:5795. doi: 10.1038/s41467-021-26046-9 (PMC8490355; doi:10.1038/s41467-021-26046-9)
Supplement: Supplementary file 1 — Supplementary Information [file 41467_2021_26046_MOESM1_ESM.pdf]

Supplementary Information

Protein Identification by Nanopore Peptide Profiling

Florian Leonardus Rudolfus Lucas[†Ψ], Roderick Corstiaan Abraham Versloot[†Ψ], Liubov Yakovlieva[‡], Marthe T. C. Walvoort[‡], Giovanni Maglia[†]*

[†]Groningen Biomolecular Sciences and Biotechnology Institute, University of Groningen, Groningen, Netherlands

[‡]*Stratingh Institute for Chemistry, University of Groningen, Groningen, Netherlands*

[Ψ]*these authors contributed equally to this work*

Corresponding Author Giovanni Maglia − Email: g.maglia@rug.nl

Table of Content

Supplementary Figure 1. Mapping of molecular weight to excluded current. 3

Supplementary Figure 2. Excluded current spectra without re-alignment. 4

Supplementary Figure 3. Excluded current re-alignment error. 5

Supplementary Figure 4. Excluded current spectra with re-alignment. 6

Supplementary Figure 5. Excluded current set against the dwell time, excluded current spectra and constructed excluded current spectra for tryptic digested proteins. 7-8

Supplementary Figure 6. Protein identification using nanopore spectrometry. 9

Supplementary Figure 7. Reproducibility of nanopore protein spectra. 10-11

Supplementary Table 1. Oligonucleotide sequences of expressed proteins. 12-13

Supplementary References. 13

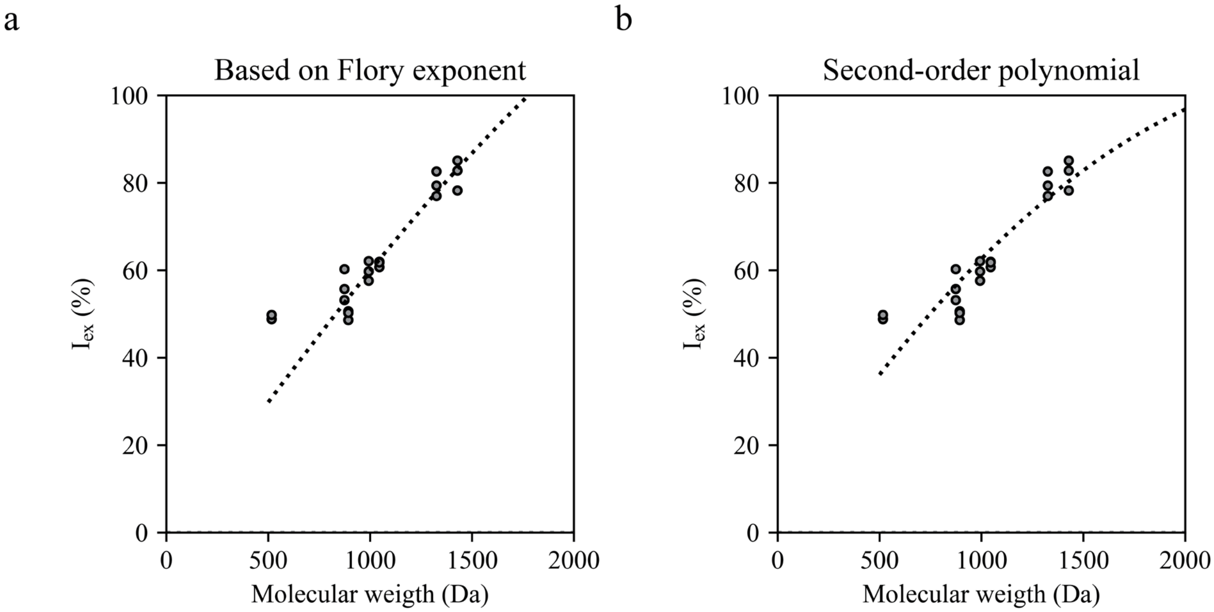

Supplementary Figure 1. Mapping of molecular weight to excluded current. A. Mass of model peptides from tryptic *Gallus-gallus* lysozyme (grey) set against the measured excluded current (%), based on 3 individual measurements. The dashed line represents a fit through the data based on the Flory exponent as described by Chavis et al.[1]:

$$I_{ex}\% = \left(1-\left(1-\left(\frac{M_p}{M_f}\right)^{\nu}\left(1-a\left(\frac{M_p}{M_f}\right)^{\gamma}\left(1-\left(\frac{M_p}{M_f}\right)^{1-\nu}\right)^{-1}\right)\right)^{-1}\right) * 100\%$$

, with model parameters: M_f = 1825.85 Da, *a* = 0.98, *v* = 0.27, *γ* = 0.0, where M_f is the mass of the smallest polymer that fills the pore volume completely, *v* is the Flory exponent, *a* and *γ* are the free parameters describing ionic binding to the polymer.[1] We excluded the datapoint at 516 Da because such model was developed for polymers and it is expected to perform poorly for small peptides. Furthermore, the last data point has a large leverage on the data. In addition, it should be noticed that the model mentioned before was developed for cylindrical nanopores, rather than for conical nanopore, such as FraC. The dashed line stops at the values that are accessible experimentally. **B.** Mass of model peptides from tryptic *Gallus-gallus* lysozyme (grey) set against the measured excluded current (%), based on 3 individual measurements. The dashed line represents an empirical second-order polynomial fit ($y=b_0+b_1*x+b_2*x^2$) through the data with b_2 = -1.33*10^{-5} , b_1 = -7.23*10^{-2}, b_0 = 3.28. Source data are provided as a Source Data file.

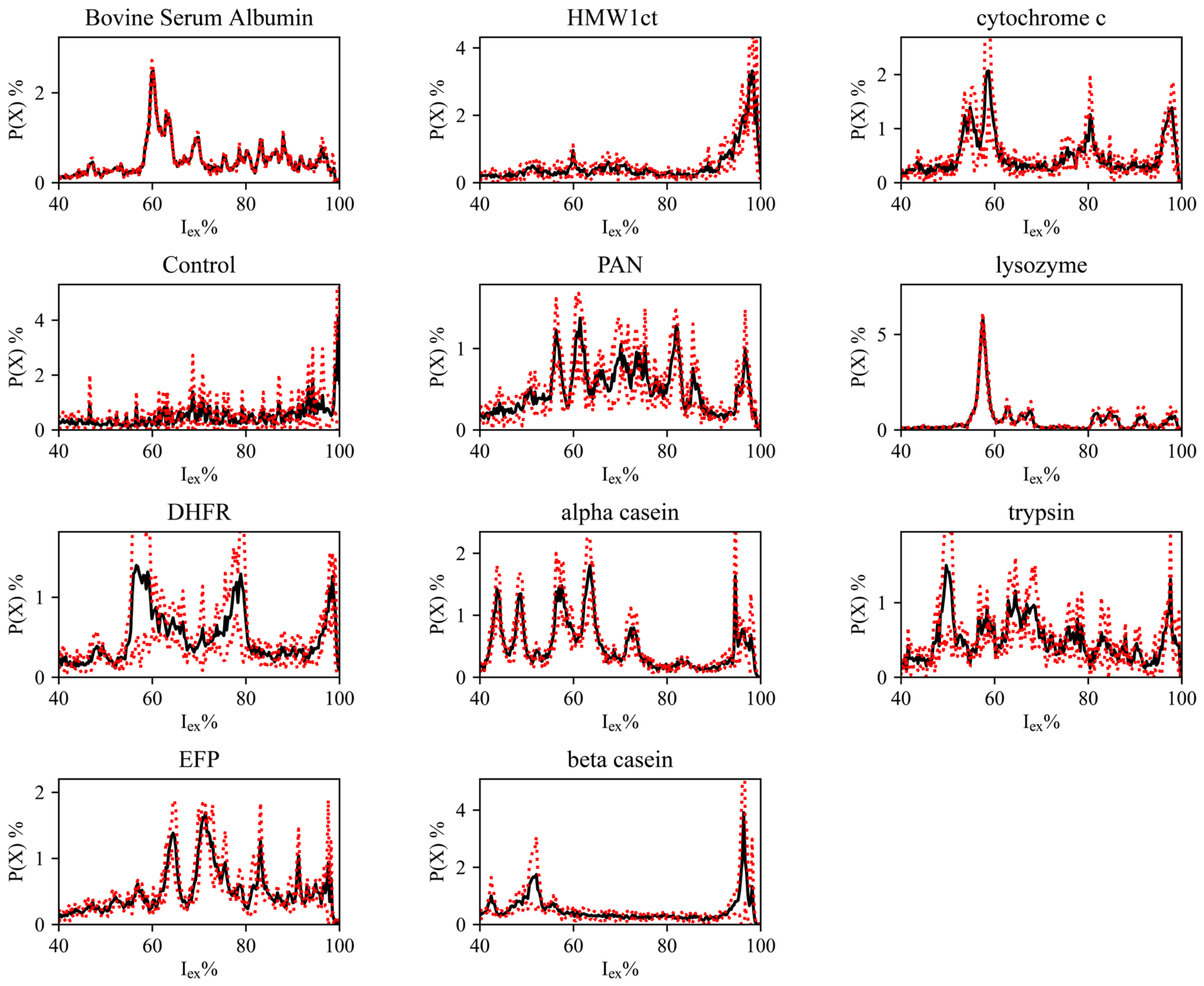

Supplementary Figure 2. Excluded current spectra without re-alignment. Resulting excluded current spectrum of 9 tryptic digested proteins and a control ("tryptic digest" of water) prior to spectral re-alignment, normalized to a total area of 100%. The solid black line represents the mean of 3 individual measurements of the same sample. The red dashed lines represent the standard deviation of 3 individual measurements. All measurements were performed in 1 M KCl buffered to pH 3.8 using 50 mM citric acid titrated with bis-tris-propane under an applied potential of -70 mV. Recording was performed at 50 kHz using an analog Bessel-filter at 10 kHz and a digital Gaussian filter of 5 kHz.

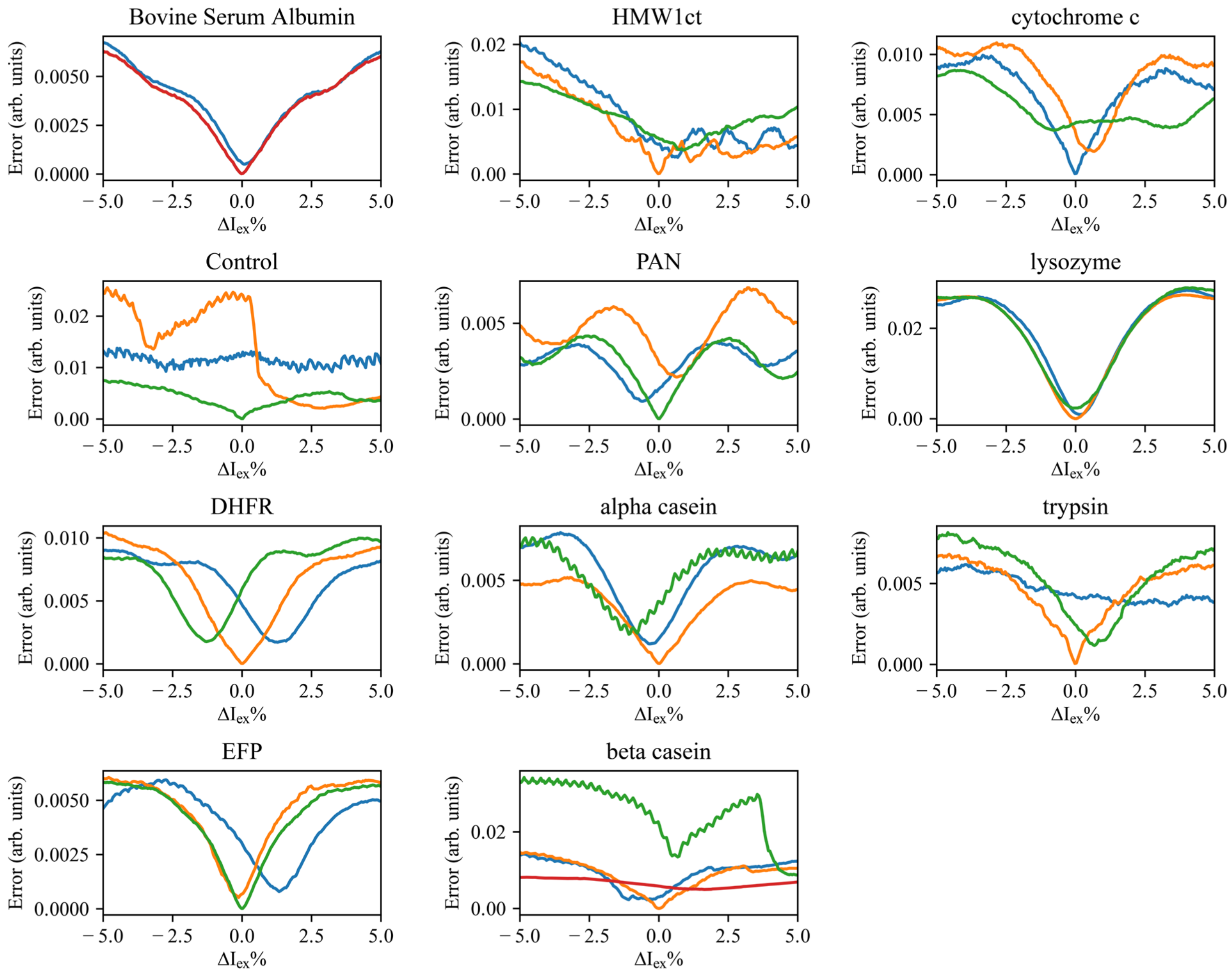

Supplementary Figure 3. Excluded current re-alignment error. Re-alignment error of 9 tryptic digested proteins and a control ("tryptic digest" of water). Each (colored) line represents a spectral alignment, the color of each line indicates a separate measurement.

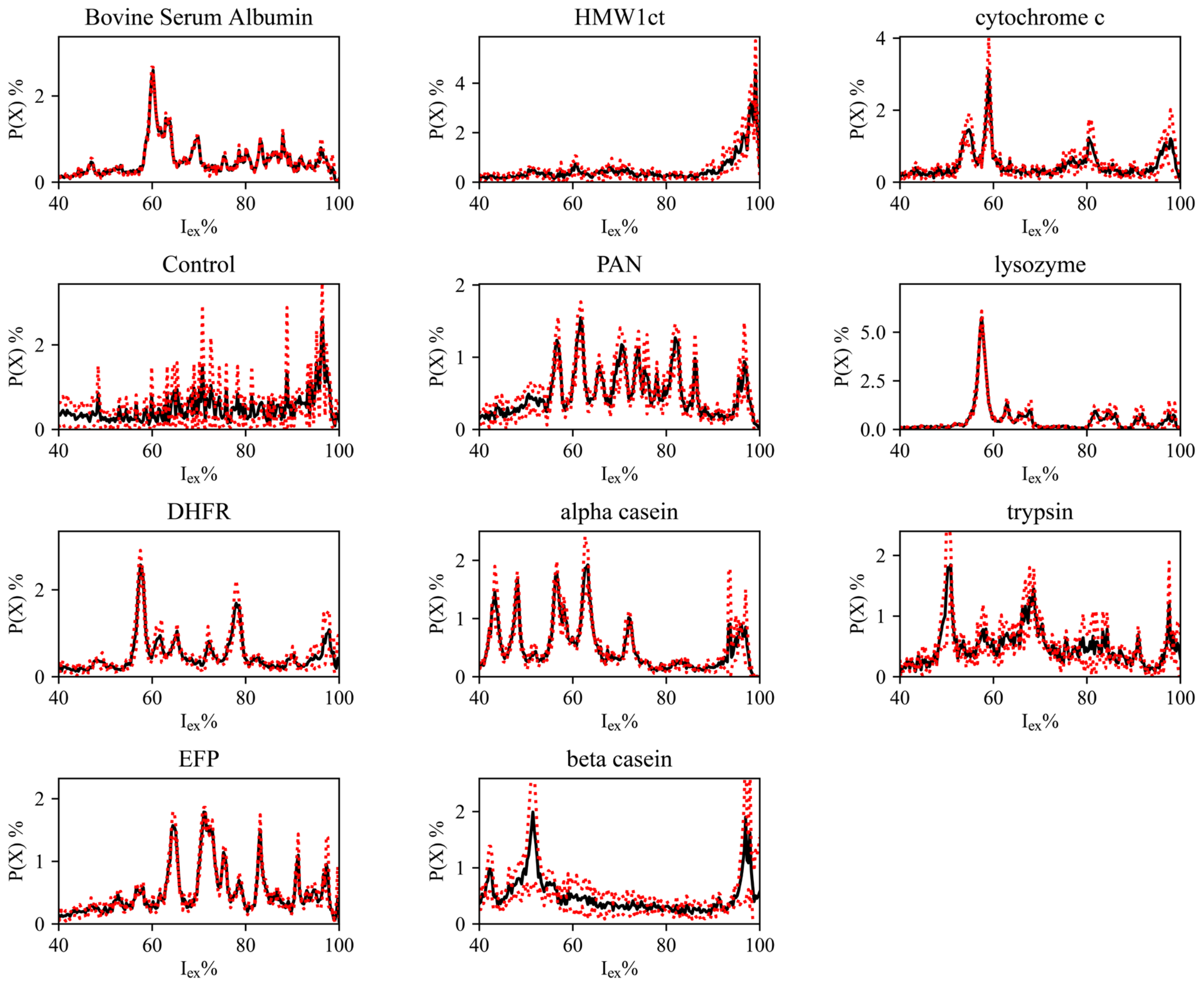

Supplementary Figure 4. Excluded current spectra with re-alignment. Resulting excluded current spectrum of 9 tryptic digested proteins and a control ("tryptic digest" of water) after spectral re-alignment, normalized to a total area of 100%. The solid black line represents the mean of 3 individual measurements of the same sample. The red dashed lines represent the standard deviation of 3 individual measurements. All measurements were performed in 1 M KCl buffered to pH 3.8 using 50 mM citric acid titrated with bis-tris-propane under an applied potential of -70 mV. Recording was performed at 50 kHz using an analog Bessel-filter at 10 kHz and a digital Gaussian filter of 5 kHz.

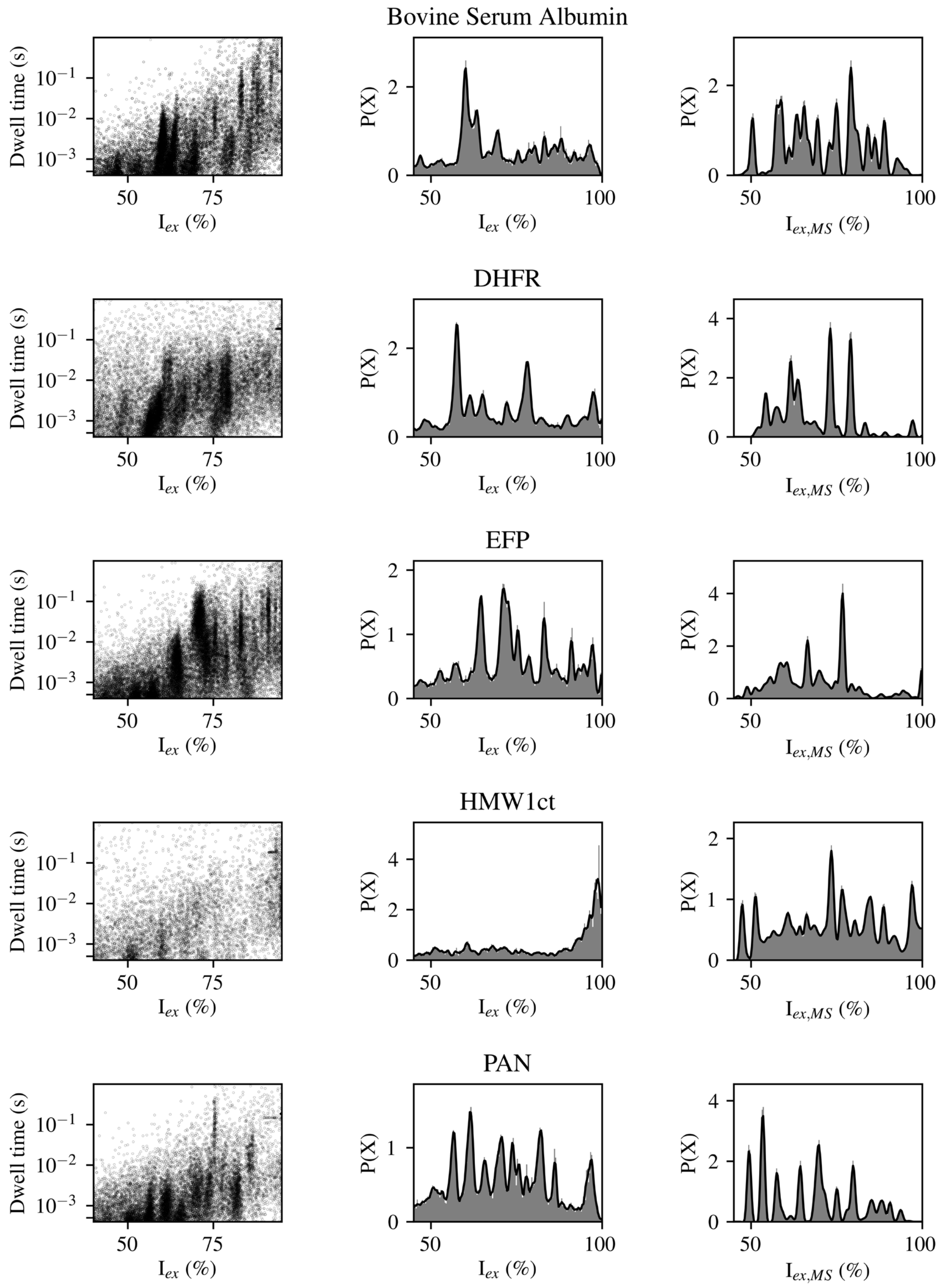

Bovine Serum Albumin
DHFR
EFP
HMW1ct
PAN
Dwell time (s)
P(X)
I$_{ex}$ (%)
I$_{ex,MS}$ (%)

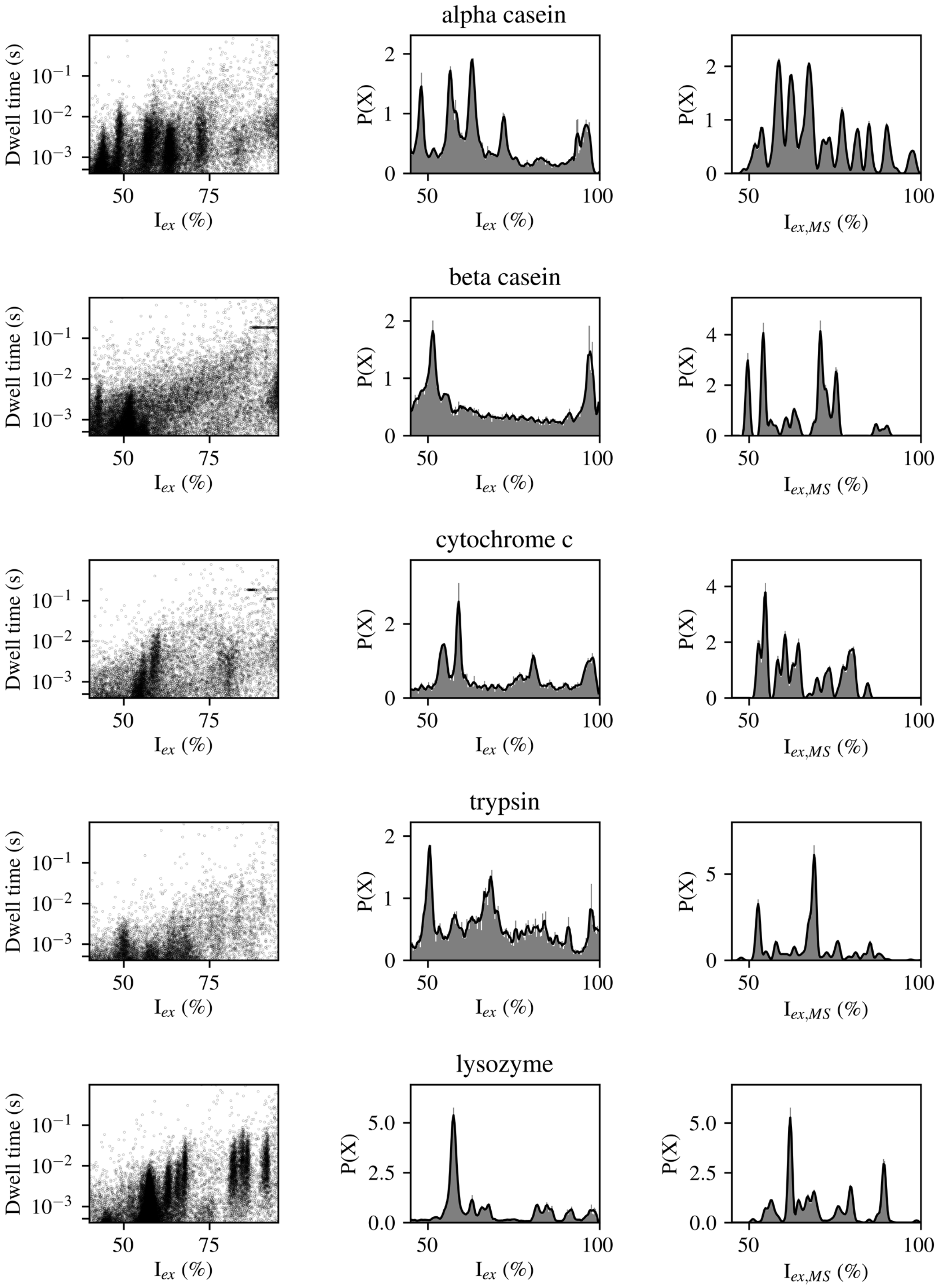

Supplementary Figure 5. Excluded current set against the dwell time, excluded current spectra and constructed excluded current spectra for tryptic digested proteins. Each row represents the data for a protein as marked with a title on the central column. The first column shows the excluded current set against the dwell time for each event. The second column shows the excluded current spectrum as measured by nanopore electrophysiology. The third column shows the predicted excluded current spectrum from mass spectrometry based results.

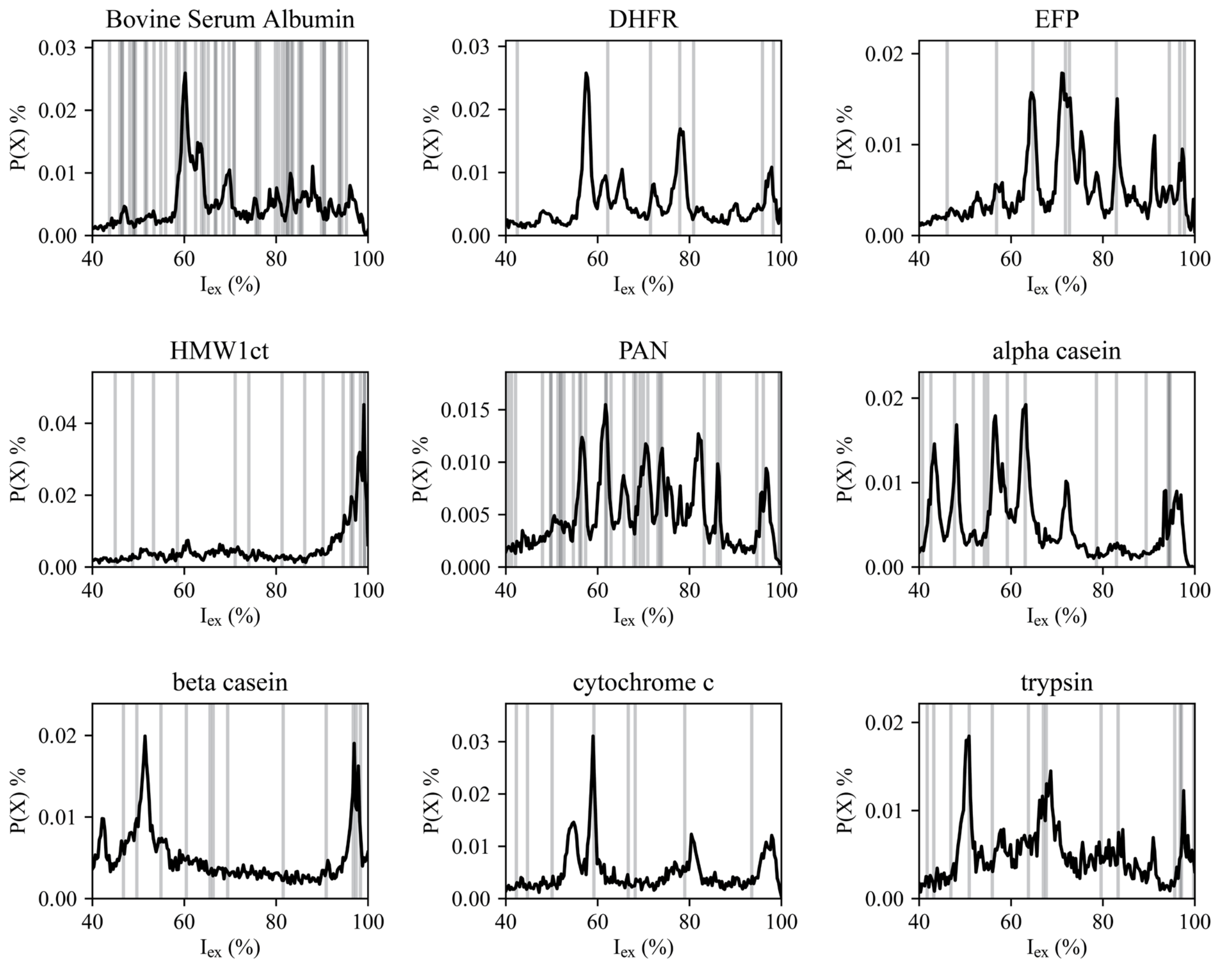

Supplementary Figure 6. Protein identification using nanopore spectrometry. Resulting baseline corrected excluded current spectra of 9 tryptic digested proteins. The vertical lines represent the peptides as predicted *in-silico*, mapped to the $I_{ex}\%$.

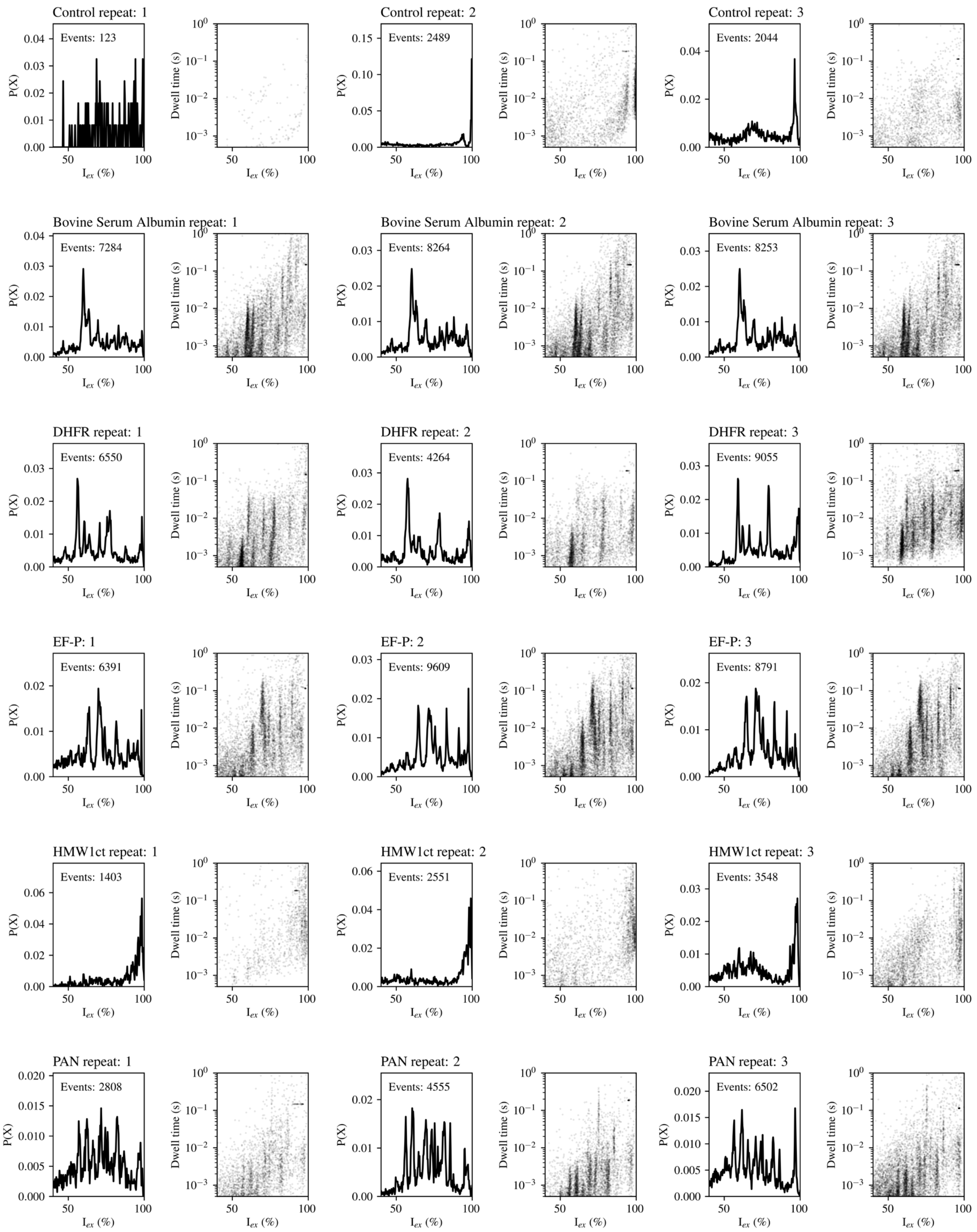

Control repeat: 1
Events: 123
Control repeat: 2
Events: 2489
Control repeat: 3
Events: 2044
Bovine Serum Albumin repeat: 1
Events: 7284
Bovine Serum Albumin repeat: 2
Events: 8264
Bovine Serum Albumin repeat: 3
Events: 8253
DHFR repeat: 1
Events: 6550
DHFR repeat: 2
Events: 4264
DHFR repeat: 3
Events: 9055
EF-P: 1
Events: 6391
EF-P: 2
Events: 9609
EF-P: 3
Events: 8791
HMW1ct repeat: 1
Events: 1403
HMW1ct repeat: 2
Events: 2551
HMW1ct repeat: 3
Events: 3548
PAN repeat: 1
Events: 2808
PAN repeat: 2
Events: 4555
PAN repeat: 3
Events: 6502
P(X)
Dwell time (s)
I_{ex} (%)

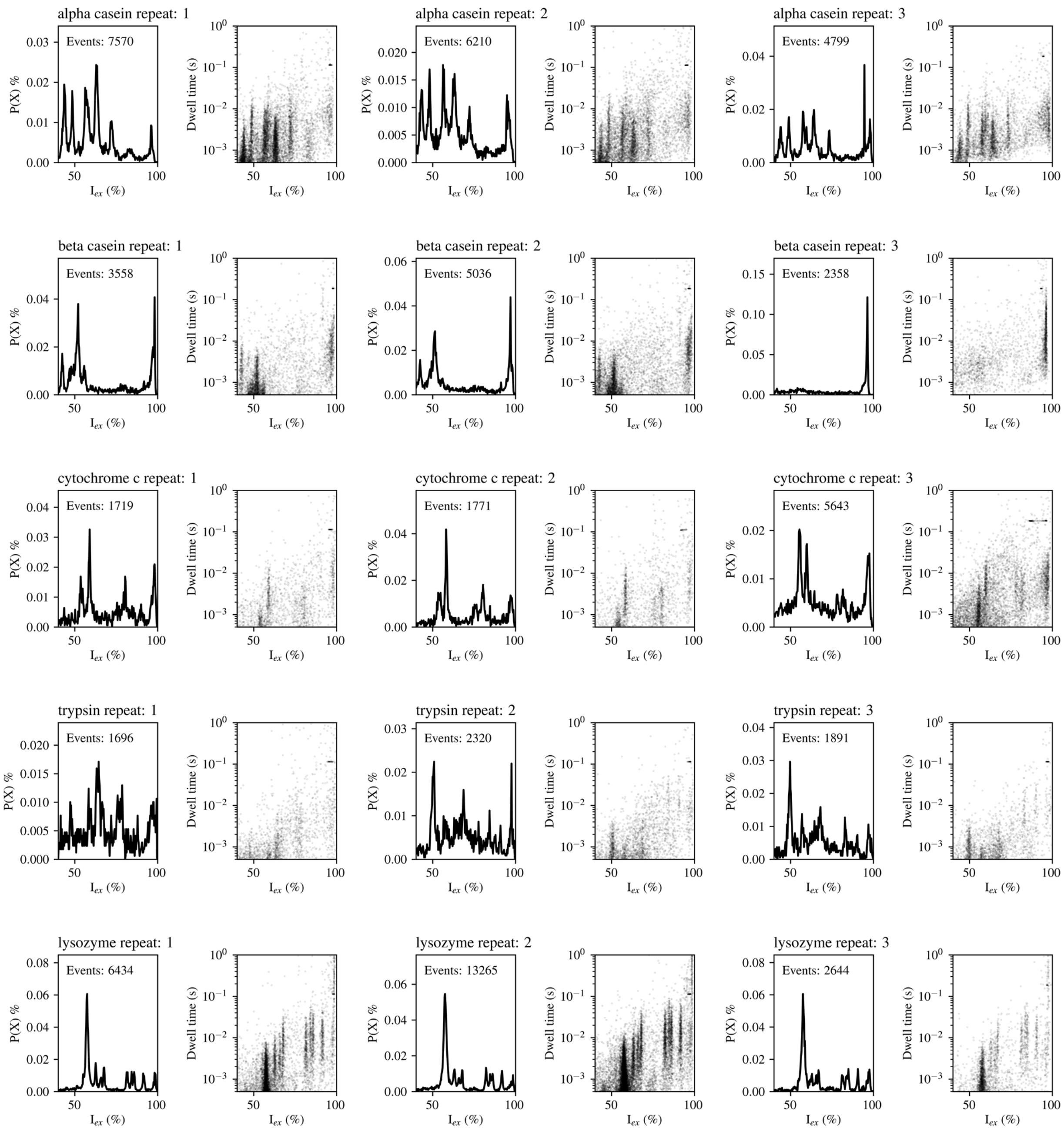

Supplementary Figure 7. Reproducibility of nanopore protein spectra. Each row presents three independent repeats for each proteolytic digest. The left panel shows the excluded current histograms with a normalized area of 100% and the right panel shows the excluded current set against the dwell time (in seconds) for all events. All measurements were performed in 1 M KCl buffered to pH 3.8 using 50 mM citric acid titrated with bis-tris-propane under an applied potential of -70 mV. Recording was performed at 50 kHz using an analog Bessel-filter at 10 kHz and a digital Gaussian filter of 5 kHz.

Fragaceatoxin C (from *Actinia fragacea*) mutant G13F G13F-FraC

ATGGCGAGCGCCGATGTCGCGGGTGCGGTAATCGACGGTGCGTTCCTGGGCTTTGACGTACTGAAAACCGTGC
TGGAGGCCCTGGGCAACGTTAAACGCAAAATTGCGGTAGGGATTGATAACGAATCGGGCAAGACCTGGACAG
CGATGAATACCTATTTCCGTTCTGGTACGAGTGATATTGTGCTCCCACATAAGGTGGCGCATGGCAAGGCGCTG
CTGTATAACGGTCAAAAAAATCGCGGTCCTGTCGCGACCGGCGTAGTGGGTGTGATTGCCTATAGTATGTCTGA
TGGGAACACACTGGCGGTACTGTTCTCCGTGCCGTACGATTATAATTGGTATAGCAATTGGTGGAATGTGCGTG
TCTACAAAGGCCAGAAGCGTGCCGATCAGCGCATGTACGAGGAGCTGTACTATCATCGCTCGCCGTTTCGCGGC
GACAACGGTTGGCATTCCCGGGGCTTAGGTTATGGACTCAAAAGTCGCGGCTTTATGAATAGTTCGGGCCACG
CAATCCTGGAGATTCACGTTACCAAAGCAGGCTCTGCGCATCATCACCACCATCACTGA

Dihydrofolate reductase (from *Escherichia coli*) DHFR

ATGGCTCACCACCACCACCACCACGGTTCGGCTATGATTTCTCTGATTGCGGCACTGGCTGTCGATCGTGTTATT
GGTATGGAAAACGCTATGCCGTGGAATCTGCCGGCTGATCTGGCGTGGTTTAAACGTAACACCCTGGACAAGC
CGGTCATTATGGGCCGCCATACGTGGGAAAGCATCGGTCGTCCGCTGCCGGGTCGCAAAAATATTATCCTGAG
CAGCCAGCCGGGCACCGATGACCGTGTGACGTGGGTTAAGAGCGTCGATGAAGCAATTGCGGCGGCAGGCGA
CGTGCCGGAAATTATGGTTATCGGCGGTGGCCGCGTTTATGAACAGTTCCTGCCGAAAGCCCAAAAGCTGTACC
TGACCCATATCGATGCAGAAGTCGAAGGTGATACGCACTTTCCGGACTATGAACCGGATGACTGGGAAAGTGT
GTTCTCCGAATTTCACGACGCCGACGCTCAGAACAGCCACTCATACTCATTCGAAATCCTGGAACGCCGTTGATA
A

proteasome-activating nucleotidase (from *Methanocaldococcus jannaschii*) PAN

ATGGCTCATCATCACCATCACCACGGGGGCGTTTTCGAAGAATTTATCTCTACTGAGTTGAAGAAGGAAAAGAA
GGCATTCACAGAGGAGTTCAAAGAAGAGAAGGAGATTAACGACAATAGCAACTTGAAGAACGATCTGTTGAA
AGAAGAATTACAAGAGAAGGCTCGTATTGCAGAGCTGGAATCACGCATTCTTAAGCTGGAGTTAGAGAAAAAA
GAGTTAGAGCGCGAAAATTTGCAGCTTGCAAAAGAAAACGAGATTTTACGTCGTGAACTTGACCGTATGCGTG
TTCCGCCTTTAATTGTAGGAACAGTGGTGGACAAAGTCGGAGAACGTAAAGTTGTGGTTAAATCCTCCACCGGA
CCATCTTTCTTAGTTAACGTGTCACATTTTGTGAACCCGGATGATCTGGCCCCTGGCAAGCGTGTATGCTTGAAT
CAACAGACTCTTACGGTTGTTGATGTATTACCGGAAAATAAGGATTACCGTGCCAAGGCGATGGAGGTGGATG
AACGTCCAAATGTGCGTTACGAGGACATCGGTGGATTAGAGAAGCAAATGCAGGAAATCCGTGAAGTAGTCG
AACTGCCGTTAAAGCACCCTGAGTTATTCGAAAAGGTCGGAATTGAACCACCTAAAGGGATTTTATTGTATGGA
CCCCCTGGGACAGGCAAAACCCTGCTGGCCAAGGCAGTTGCGACGGAAACTAACGCAACCTTTATTCGCGTGG
TCGGTTCAGAGCTTGTTAAGAAGTTCATCGGAGAAGGAGCGAGCTTAGTGAAGGATATTTTTAAGCTGGCCAA
GGAGAAGGCTCCCTCGATTATCTTTATCGATGAGATCGATGCGATCGCAGCCAAGCGTACGGATGCATTAACG
GGTGGGGATCGTGAAGTACAACGCACACTGATGCAGCTTCTTGCCGAAATGGATGGGTTCGATGCACGTGGAG
ACGTCAAGATCATTGGCGCAACAAACCGTCCAGATATCCTTGATCCAGCGATCCTTCGCCCAGGTCGTTTCGACC
GCATTATCGAGGTCCCGGCACCGGACGAAAAGGGCCGCCTTGAGATTTTGAAAATTCATACACGCAAAATGAA
CTTGGCAGAGGATGTAAACTTGGAAGAAATTGCTAAGATGACTGAGGGGTGCGTAGGTGCAGAACTGAAGGC
TATTTGCACGGAGGCCGGAATGAATGCAATCCGTGAACTTCGTGATTATGTAACGATGGACGATTTTCGCAAAG
CAGTTGAGAAAATCATGGAGAAAAAGAAGGTTAAGGTAAAAGAGCCAGCCCATCTGGACGTACTGTATCGTTG
ATAA

high molecular weight adhesin 1 (from *Haemophilus influenzae*) HMW1ct

ATGGCACATCACCACCACCATCACGTGTGGACCGCAAACTCAGGCGCACTGACCACGCTGGCGGGTTCTACCAT
CAAAGGCACGGAATCGGTTACGACCTCATCCCAATCGGGTGACATCGGCGGCACCATTAGTGGCGGTACGGTC
GAAGTGAAAGCGACCGAATCCCTGACCACGCAGAGCAACTCTAAAATCAAAGCGACCACGGGCGAAGCCAAT
GTTACCAGCGCAACCGGCACGATTGGCGGCACCATCTCTGGTAACACCGTTAATGTCACGGCAAACGCTGGTG
ATCTGACCGTGGGCAACGGTGCAGAAATTAATGCTACCGAAGGTGCAGCAACGCTGACCACCAGCAGCGGTAA

ACTGACCACGGAAGCCAGTTCCCATATTACCTCAGCAAAAGGCCAGGTCAACCTGTCGGCTCAAGACGGCTCAG
TGGCGGGTTCGATCAACGCAGCTAATGTTACCCTGAATACCACGGGCACGCTGACCACGGTCAAAGGTAGTAA
CATTAATGCCACCTCCGGTACGCTGGTGATCAACGCAAAAGATGCTGAACTGAATGGCGCGGCCCTGGGTAAC
CACACCGTGGTTAATGCGACGAACGCCAATGGCAGTGGTTCCGTGATTGCGACCACGTCATCGCGTGTTAACAT
CACCGGCGACCTGATTACGATCAACGGTCTGAACATCATCAGCAAAAACGGCATCAACACCGTTCTGCTGAAAG
GTGTTAAAATCGATGTCAAATACATCCAGCCGGGCATCGCTTCTGTGGACGAAGTTATTGAAGCGAAACGCATC
CTGGAAAAAGTGAAAGATCTGAGTGACGAAGAACGTGAAGCGCTGGCCAAACTGGGTGTCAGCGCCGTGCGC
TTTATTGAACCGAACAATACCATCACGGTTGATACCCAAAATGAATTCGCAACCCGTCCGCTGAGCCGCATTGTC
ATCTCTGAAGGCCGTGCCTGCTTTAGTAACTCTGACGGTGCTACGGTTTGCGTGAACATCGCTGATAATGGTCG
T

elongation factor P (from *Pseudomonas aeruginosa*) EF-P
ATGAAAACCGCTCAAGAGTTCCGCGCCGGCCAGGTTGCCAACATCAATGGCGCTCCCTGGGTCATCCAGAAGG
CCGAGTTCAACAAGTCCGGCCGTAACGCTGCCGTCGTCAAGATGAAGCTGAAGAACCTGCTGACCGGCGCCGG
TACCGAGACCGTGTTCAAGGCCGACGACAAGCTGGAGCCGATCATCCTCGATCGCAAGGAAGTGACCTACTCC
TACTTCGCCGACCCGCTGTACGTCTTCATGGACAGCGAGTTCAACCAGTACGAGATCGAGAAAGACGATCTGGA
AGGCGTGCTGACCTTCATCGAAGACGGCATGACCGACATCTGCGAAGCCGTGTTCTACAACGACAAGGTGATC
TCGGTAGAGCTGCCGACCACCATCGTTCGCCAGATCGCCTACACCGAGCCGGCCGTCCGCGGCGACACCTCGG
GCAAGGTGATGAAGACCGCGCGCCTGAACAACGGCGCCGAGTTGCAGGTTTCCGCGTTCTGCGAAATCGGCGA
CTCGATCGAGATCGATACCCGCACCGGCGAGTACAAGTCCCGCGTCAAGGCCTGA

Supplementary Table 1. Oligonucleotide sequences of expressed proteins. This table contains the oligonucleotide sequences used to express fragaceatoxin C mutant G13F, dihydropholate reductase, proteasome-activating nucleotidase, high molecular weight adhesin 1, and elongation factor P.

Supplementary References

(1) Chavis, A. E.; Brady, K. T.; Hatmaker, G. A.; Angevine, C. E.; Kothalawala, N.; Dass, A.; Robertson, J. W. F.; Reiner, J. E. Single Molecule Nanopore Spectrometry for Peptide Detection. *ACS Sensors* **2017**, *2* (9), 1319–1328. https://doi.org/10.1021/acssensors.7b00362.
